# Detection and Localization of Partial Discharge in Connectors of Air Power Lines by Means of Ultrasonic Measurements and Artificial Intelligence Models

**DOI:** 10.3390/s21010020

**Published:** 2020-12-22

**Authors:** Vykintas Samaitis, Liudas Mažeika, Audrius Jankauskas, Regina Rekuvienė

**Affiliations:** Prof. K. Baršauskas Ultrasound Research Institute, Kaunas University of Technology, K. Baršausko St. 59, LT-51423 Kaunas, Lithuania; liudas.mazeika@ktu.lt (L.M.); audrius.jankauskas@ktu.lt (A.J.); regina.rekuviene@ktu.lt (R.R.)

**Keywords:** partial discharge, bushing insulators, ultrasonic localization, ultrasonic time-of-flight evaluation, machine learning, deep learning

## Abstract

According to the statistics, 40% of unplanned disruptions in electricity distribution grids are caused by failure of equipment in high voltage (HV) transformer substations. These damages in most cases are caused by partial discharge (PD) phenomenon which progressively leads to false operation of equipment. The detection and localization of PD at early stage can significantly reduce repair and maintenance expenses of HV assets. In this paper, a non-invasive PD detection and localization solution has been proposed, which uses three ultrasonic sensors arranged in an L shape to detect, identify and localize PD source. The solution uses a fusion of ultrasonic signal processing, machine learning (ML) and deep learning (DL) methods to classify and process PD signals. The research revealed that the support vector machines classifier performed best among two other classifiers in terms of sensitivity and specificity while classifying discharge and surrounding noise signals. The proposed ultrasonic signal processing methods based on binaural principles allowed us to achieve an experimental lateral source positioning error of 0.1 m by using 0.2 m spacing between L shaped sensors. Finally, an approach based on DL was suggested, which allowed us to detect a single PD source in optical images and, in such a way, to provide visual representation of PD location.

## 1. Introduction

According to the EU study on electricity supply disruptions, in the period 2010–2014, up to 850 GWh of electricity annually is not supplied to the consumers, which caused a lost value up to EUR 25 billion per year to the commercial users [1]. The vast majority of these disruptions occur due to the problems in distribution grids that are caused by the failure of insulation of the conductors, such as presence of voids and cracks. This leads to a partial discharge (PD), which can be defined as a localized breakdown of the insulation under the stress of high voltage [2]. In case of PD, the bridge at the certain part of insulation is created which transports the electric charge between two electrodes. PD tend to develop over time, which causes progressive damage to high voltage (HV) assets and leads to false operation of the distribution grid. Therefore, early-stage PD detection, localization and monitoring are significant tasks to ensure safe and reliable supply of the electric power.

As the location and type of discharge may impact HV assets differently, the positioning of the PD source is of considerable practical interest. In the event of the PD, the energy is transmitted into the surrounded media as electromagnetic waves, heat, light and acoustic waves, so it can be detected utilizing various sensing technologies [3,4]. Acoustic measurements have shown several advantages in PD detection over the existing techniques, such as immunity to electromagnetic interference, ability to localize discharge sources, easy and non-invasive deployment on-site. Ultrasonic PD localization technologies use sensor arrays of different configurations to detect sound waves generated by discharge and measure time of flight between subsequent channels [5,6,7]. In such a way, an approximate position of the source can be determined. Various sensor array configurations, such as circular and cross-shaped, have been investigated by different research groups, showing PD source angular positioning errors of approximately 5° [8,9,10]. Among the signal processing approaches, the wavelet transform, Hilber-Huang transform and empirical mode decomposition are frequently used to describe PD signals [11,12,13]. In most of the researches, the spectral signal features are exploited for PD detection, however other works show that time domain analysis can be beneficial for PD detection, especially when the repetition rate and signal reverberations within the substation are important [14]. For the PD classification tasks, recent advances in pattern recognition techniques based on artificial intelligence have been widely implemented [15,16]. Both unsupervised [17,18,19] and supervised [20,21,22] learning techniques are used to group PD signals according to their statistical similarities. For example, Lewin et al. [23] used DBSCAN and t-SNE clustering along with wavelet denoising to discriminate PD signals arriving from multiple sources. Contin et al. [24] presented K-means based clustering method to separate PD signals in case of multiple active sources. Li et al. [25] used two back propagation neural networks for partial discharge recognition in gas insulated switchgear. Choi et al. [26] tested various ML methods like bagging, k-nearest neighbor, support vector machines and linear discriminant analysis to detect cap damage of porcelain insulators using frequency response functions. An increasing number of researches that use deep learning (DL) neural networks for PD classification can be noticed recently. Florkowski [27] used convolutional neural networks (CNN) to detect deterioration of electrical insulation from the phased resolved PD images. In contrast to machine learning methods, DL do not require feature extraction, can handle large datasets and provide better accuracy [28,29].

Most of the aforementioned advances in sensing and PD classification are applied to detect, de-noise and localize discharges in oil-filled transformer tanks, where discharge-induced acoustic signals propagate in oil with relatively low attenuation. For example, recent research of Hamidreza et al. [30] show that by using the time reversal approach, the PD source can be localized with superior resolution of λ/10 using one to few sensors. On the other hand, its estimated that up to 17% of total transformer failures are caused by faulty bushings in air power lines [31]. These can be produced using resin-bonded paper, resin-integrated paper or resin-impregnated synthetic technologies, which can be susceptible to PD. Ultrasonic assessment of PD in transformer bushings can be completed by using contactless techniques in open-air. This introduces additional challenges, such as high transmission losses of acoustic signals, noise and multiple reflections within transformer substation, relatively short inspection distances and increased discharge source positioning errors. Among the attempts to detect and localize corona discharge, Dong et al. [32] used a fusion of ultrasonic measurement and ultra-violet imaging to visualize the corona source locations in optical views with an angular error of 5.32% at 30 m distance.

In this research, hybrid an approach to detect, localize and visualize corona discharge source is presented. The technique proposed in this study is based on the fusion of ultrasonic and optical data using machine learning and deep learning methods, which enable intuitive discharge visualization in a real-scene environment. The technique uses ultrasonic measurements to detect and localize the source of discharge, while the source itself is identified and emphasized with optical camera by using deep learning methods. The proposed methods approach demonstrates increased sensitivity to PD and low lateral source positioning errors up to 0.1 m. In comparison with similar work conducted by Dong et al. [23], the proposed technique offers a cost-effective way to detect PD without using an expensive ultra-violet imaging equipment employing only three acoustic sensors for PD localization instead of 31. The obtained solution provides lower positioning errors, due to specific arrangement of ultrasonic sensors with optimized inter-element distance, successful implementation of machine learning models to filter random noise signals and precise time of flight (ToF) measurements. The workflow of the proposed approach can be described as follows. At first, the proposed technology identifies discharge signals and differentiates them from random noise using different machine learning methods like Support Vector Machines (SVM), Naïve Bayes (NB) and Linear Discriminant Analysis (LDA) classifiers. A comprehensive study based on Pearson correlation, *t*-test analysis and Mahalanobis distance evaluation is performed here in order to extract features representing discharge signals. The identified discharge signals are then analyzed with ultrasonic signal processing methods to estimate the spatial source position in open-air. The proposed solution uses only three ultrasonic sensors arranged in an L shape to detect and localize discharge location, while the inter-element distance is optimized to achieve the desired accuracy and compact design of the system. The detected position of discharge source is provided to pan-tilt servo motors which control the movement of an optical camera. Then, deep learning networks are trained to detect bushing insulators in optical images. The final output of the proposed technique is an optical image with detected suspicious bushing elements.

This paper is organized as follows: In the first chapter, the ML methods and discharge feature extraction are described for discriminating discharge signals from noise. Then the ultrasonic technique to detect source angular position in two orthogonal planes is presented and verified with appropriate experiments. Finally, DL methods are described for transformer bushing detection in optical image at the direction of PD source.

## 2. Architecture of Proposed Discharge Detection Technique

**Figure 1:**
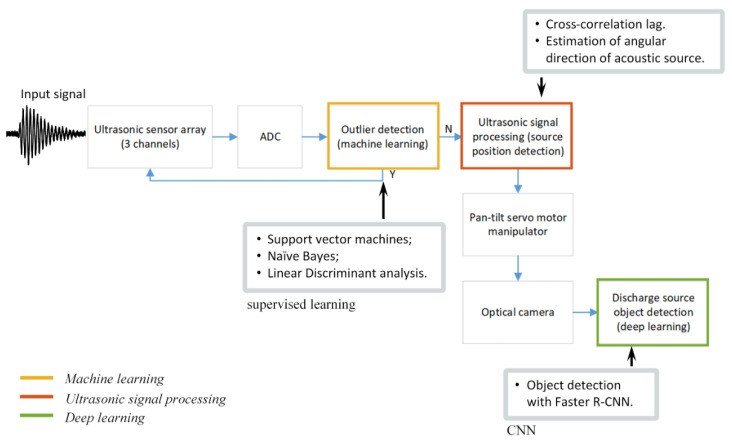
The architecture of proposed discharge detection system.

The architecture of proposed PD detection system is presented in Figure 1. It consists of hardware parts that detect, digitize and process PD signals and signal processing—part which is responsible for discharge signal identification—source localization and recognition.

**Figure 2:**
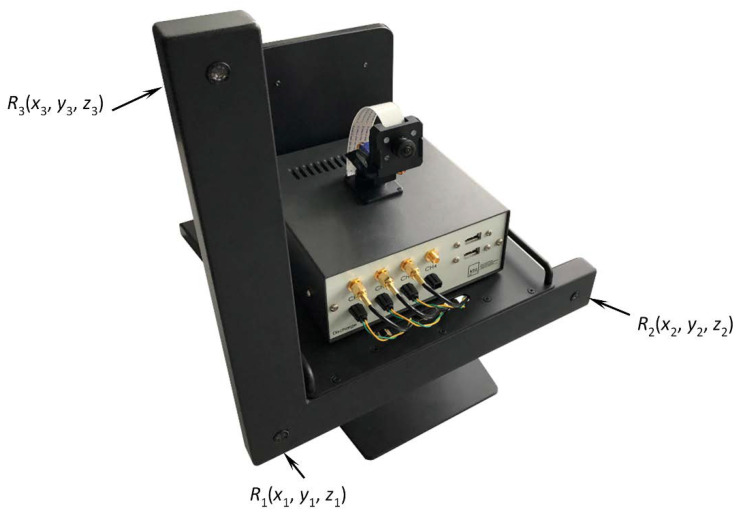
Front view of the proposed partial discharge detector.

The system was designed for discharge signal detection in open-air at 40 kHz frequency with 3 sensors arranged in an L shape (see Figure 2). Such frequency characteristics of the system was selected taking into the account attenuation of sound in air (−2.6 dB/m, at 60% relative humidity and 40 kHz) and ensuring operating distance of the device up to 30 m. Such design of the system ensures non-invasive inspection of transformer bushings and reduces noise that is created by HV assets since all measurements are taken at least from a distance of 5 m. Commercially available open-structure air coupled 40 kHz ultrasonic transducers (MA40S4R produced by Murata Manufacturing Co., Ltd. (Kyoto, Japan) with directivity of 80°, sensitivity of −63 dB and bandwidth of 5 kHz at −6 dB level) were selected for discharge signal acquisition. The amplification circuit was manufactured using low noise operational amplifier which provides 50 dB gain (at 40 kHz) and input voltage noise of $5.8\;\mathrm{nV}/\sqrt{\mathrm{Hz}}$. The 20 MHz 12bit 4-channel analogue to digital converter was used to digitize the PD signals. The front view of the proposed PD detector is presented in Figure 2.

The signal processing part of the system implements ML algorithms to discriminate signals originating from noise. These ML algorithms act as firewall, so further signal processing is skipped if the signal is found to be originating from surrounding noise. If the received signal is originated from the PD, then the ultrasonic signal processing methods are used to determine spatial location of PD source. Finally, to recognize discharging asset, DL convolutional neural networks are implemented, which detect suspicious discharging asset in optical images. In the few following sections, the discharge signal identification, localization and recognition techniques will be discussed in further details.

## 3. A technique to Identify Discharge Signals

To complete the purpose of discriminating PD signals from surrounding noise, three machine learning classifiers were implemented and tested, named Support Vector Machines (SVM) with Radial Basis Function (RBF) kernel, Naïve Bayes (NB) and Linear Discriminant Analysis (LDA). The dataset of discharge-induced signals was acquired using laboratory corona discharge simulator. The PD source was a needle–needle electrode separated at 230 mm distance. The emitted ultrasound signals were collected at 40 kHz with sampling of 1 MS/s, maintaining 10 m distance from source and sensors. In total, 150 signals were acquired that represent the PD data. The noise signals were recorded from the surroundings of the transformer sub-station, registering signals every 12 min for period of 24 h. This resulted in 120 signals representing acoustical noise in the sub-station. To represent each of the dataset, nine quantitative ultrasonic time domain features were evaluated: mean absolute value, variance, simple square integral, kurtosis, root mean square, average amplitude change, difference absolute standard deviation, modified mean absolute value and maximum fractal length [33,34]. In this research, time domain features were investigated only, as the proposed system incorporates narrowband sensors, limiting the spectrum of the received signals. The mean absolute value can be defined as the average of absolute value of the signal:

(1) $$M_{\mathrm{AV}}=\frac{1}{N}\overset{N}{\underset{i=1}{\sum }}\left|x_{i}\right|,$$

where $x_{i}$—is the magnitude of the signal at discrete time instance, N—is the length of the signal. Variance is expressed as squared sum of instantaneous values:

(2) $$v=\frac{1}{N-1}\overset{N}{\underset{i=1}{\sum }}{\left(x_{i}\right)}^{2}.$$

Simple squared integral is the summation of signal square values, without consideration of signal length:

(3) $$S=\overset{N}{\underset{i=1}{\sum }}{\left(x_{i}\right)}^{2}.$$

Kurtosis define distribution of the signal relative to normal distribution as:

(4) $$K=\frac{\sum_{i=1}^{N}{\left(x_{i}-\bar{x}\right)}^{4}}{\sigma^{4}},$$

where $\bar{x}$ is mean value of x, σ—is the standard deviation. Root mean square is another feature that defines square root of mean square as:

(5) $$R=\sqrt{\frac{1}{N}\overset{N}{\underset{i=1}{\sum }}{\left(x_{i}\right)}^{2}}.$$

Average amplitude change is the average magnitude change between neighboring instantaneous values of the signal expressed as:

(6) $$A=\frac{1}{N}\overset{N-1}{\underset{i=1}{\sum }}\left|x_{i+1}-x_{i}\right|.$$

Difference absolute standard deviation is an average amplitude change related parameter that is defined as the square root of magnitude change square:

(7) $$D=\sqrt{\frac{\sum_{i=1}^{N-1}{\left(x_{i+1}-x_{i}\right)}^{2}}{N-1}}.$$

Modified mean absolute value uses a weight window function in addition to Equation (1) as:

(8) $$M_{\mathrm{MAV}}=\frac{1}{N}\overset{N}{\underset{i=1}{\sum }}\left|x_{i}\right|w_{i},\;w_{i}=\{\begin{array}{cc} 1, & if\;0.25N\leq i\leq 0.75N\\ 0.5, & otherwise \end{array},$$

here $w_{i}$ is the weight coefficient. Finally, maximum fractal length is defined as:

(9) $$M_{F}=\log_{10}\left(\sqrt{\overset{N-1}{\underset{i=1}{\sum }}{\left(x_{i+1}-x_{i}\right)}^{2}}\right).$$

The aforementioned quantitative measures have been applied to datasets representing discharge and noise signals. All features were standardized, making the mean value of each feature equal to 0 and variance of 1 as:

(10) $$X_{k}=\frac{x_{k}-{\bar{x}}_{k}}{\sigma_{k}},$$

where $X_{k}$ is the normalized feature vector, $x_{k}$ is the initial feature vector, ${\bar{x}}_{k}$—is the mean value of the feature vector $x_{k}$, $\sigma_{k}$—is the standard deviation of feature vector $x_{k}$, k=1, 2,…m, where m is total number of available features.

As the large number of features can reduce the classification accuracy, redundant features were removed using Mahalanobis distance evaluation, *t*-test, Pearson correlation and principal component analysis. A two sample *t*-test can be formulated as follows:

(11) $$t=\frac{{\bar{x}}_{1}-{\bar{x}}_{2}}{\sqrt{s^{2}\left(\frac{1}{n_{1}}+\frac{1}{n_{2}}\right)}},\;s^{2}=\frac{\sum_{i=1}^{n_{1}}{\left(x_{i}-{\bar{x}}_{1}\right)}^{2}+\sum_{j=1}^{n_{2}}{\left(x_{j}-{\bar{x}}_{2}\right)}^{2}}{n_{1}+n_{2}-2},$$

where ${\bar{x}}_{1}$, ${\bar{x}}_{2}$—are sample means, $s^{2}$—is the sample variance, $n_{1},\;n_{2}$—are the sample sizes, t—is a Student t quantile with $n_{1}+n_{2}-2$ degrees of freedom. The Mahalanobis distance is expressed as a distance from vector to a distribution with mean and covariance as:

(12) $$d=\;\sqrt{\left(x-\mu \right)\sum_{}^{-1}{\left(x-\mu \right)}^{\prime }},$$

where x—is a vector from which the distance is being evaluated, μ—is the distribution mean.

The statistical evaluation of each of the abovementioned features is summarized in Table 1. The aim of such evaluation was to extract statistically significant features that can be further used for training and testing of machine learning models. The null hypothesis $h_{0}$ in the *t*-test was formulated as: “there is no significant difference between the discharge induced signal and random noise”. The *p*-value was calculated with the confidence interval of 95%, meaning that *p*-values higher than 5% will reject the null hypothesis. In Table 1, the $h_{0}=0$ means that the null hypothesis was rejected during the *t*-test analysis. The last row of the Table 1 represents the Pearson correlation value between discharge and noise dataset.

**Figure 3:**
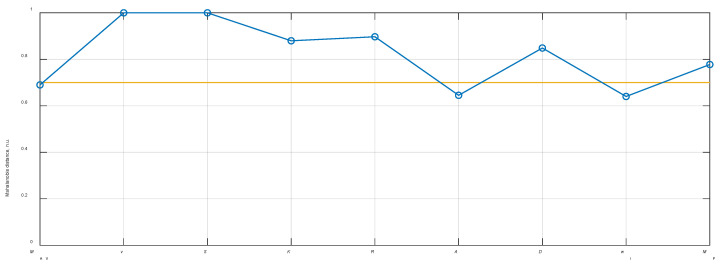
The normalized Mahalanobis distance between different statistical features of the discharge and random noise vectors (in blue) and the threshold level of 0.7 (orange) which was used to select the significant features.

The results presented in Table 1 demonstrate that there are six out of nine features that are statistically significant according to the *t*-test analysis named: variance (***v***); simple squared integral (*S*); Kurtosis (*K*); root mean square (*R*); difference absolute standard deviation (*D*); maximum fractal length (*M*_F_). In all cases, there was no significant correlation between feature vectors of noise and discharge datasets (normalized correlation around 0.2 was considered as very low), hence only the *t*-test and *p*-values were used as criteria to select significant features. The normalized Mahalanobis distance evaluation demonstrated six significant features at the pre-defined threshold of 0.7. The results of Mahalanobis distance evaluation correlated well with the *t*-test analysis, showing the same significant feature vectors. The normalized values of the Mahalanobis distance for each statistical feature are illustrated in Figure 3.

In order to further minimize the number of statistical features, principal component analysi (PCA) was performed with an intrinsic dimension of 2, indicating following most significant features for classification task: kurtosis (*K*) and difference absolute standard deviation (*D*).

**Figure 4:**
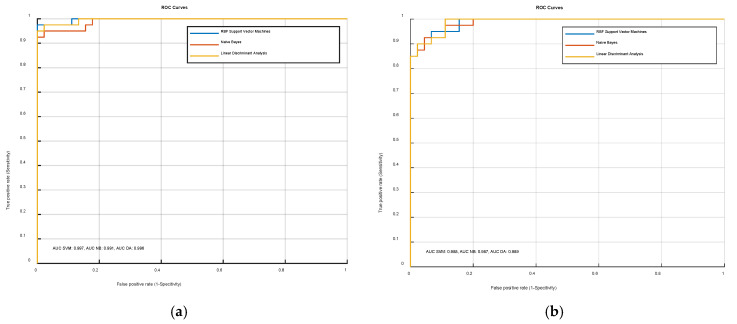
The comparison of receiver operating curve (ROC) curves using different feature vectors for training and testing of the three classifiers: (**a**) ROC in case of 6 statistically significant features; (**b**) ROC in case of 2 statistical features selected using PCA.

The abovementioned classifiers (SVM, NB, LDA) were trained and tested for both cases: using all six statistical features identified with Mahalanobis distance evaluation and only two features, identified with PCA. The results demonstrated that specificity, sensitivity and area under the receiver operating curve (ROC) were similar for all classifiers either using all six or only two feature vectors. This means that kurtosis and difference absolute standard deviation holds most of the variance in the entire dataset. The ROC curves in the case of six features and two features (kurtosis and difference absolute standard deviation) for all three classifiers are illustrated at Figure 4. The comparison between the sensitivity and specificity of three selected classifiers in the case of six and two feature vectors can be seen in Table 2.

**Figure 5:**
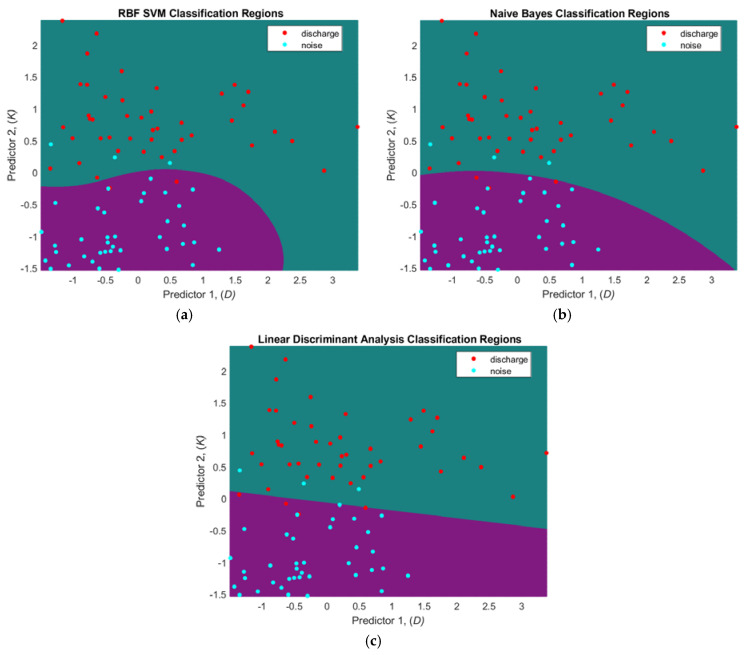
The classification boundaries of discharge and noise datasets obtained using two feature vectors and following classifiers: (**a**) RBF SVM; (**b**) Naïve Bayes (NB); (**c**) Linear Discriminant Analysis (LDA).

The results presented both in Figure 4 and Table 2 indicate that classification is accurate in both cases, either using six or two feature vectors. In case of six feature vectors, the area under the ROC curve (AUC) is 99.7% for SVM, 99.1% for NB and 99.6% for LDA classifiers. In contrast, the two-feature vector provides AUC of 98.8% for SVM, 98.7% for NB and 98.9% for LDA. The results in Table 2 indicate, that the RBF SVM classifier provides the best performance among all the classifiers in terms of sensitivity and specificity. The classification boundaries for all three classifiers in the case of the two-feature vector space is presented in Figure 5.

The results presented here demonstrate that the classification between the discharge signal and surrounding noise can be performed quite accurately. These results need to be further verified, as the discharge signals were collected using a laboratory discharge emulator. It is expected that the performance of the classifiers will slightly degrade going into the in-situ discharge measurements. The RBF SVM classifier overall demonstrated best performance, since classification boundary for the dataset appeared to be non-linear. According to the architecture of the proposed discharge detector, the ML methods presented in this chapter act as an outlier detector with the aim to filter random noise signals from further processing. If the signal is identified as a discharge, the ultrasonic localization algorithms presented in following chapter are used then to localize the source position in open space.

## 4. Ultrasonic Localization of PD Source

### 4.1. Principle of Measurement Method

Proposed acoustic noise localization method assumes that discharge is generating acoustic signal, which is recognized and received by the ultrasonic measurement system. In general, the partial discharge position measurement method is based on a binaural principle. According to this principle, it is necessary to measure the ultrasound propagation time from the source to the few receivers. The coordinates of the source can be calculated by using measured time delay and ultrasound velocity. To implement the binaural method, in this case we used three sensors arranged in an L shape (see Figure 2). Other authors suggest three main configurations of sensors for PD detection: cross, circular and square [10]. Usually, these set-ups use at least nine sensors. Xie et al. [9,10] showed acoustic comparison of the three abovementioned sensor arrangements, demonstrating that the circular array has the lowest mean positioning error of 4.5 cm at distance of 1 m. The cross-shaped arrangement demonstrated worst mean positioning accuracy of 6.2 cm. This results in a lateral source positioning error of approximately 5%. Another research conducted by Li et al. [8] used cross-shaped array of 13 elements and demonstrated source positioning error less than 5°. Finally, Dong et al. used 31 sensors in a double-helix configuration and achieved an error of 5.32% at 30 m distance. In our approach, the L-shaped sensor arrangement with three sensors was selected with the aim to use the minimum possible amount of transducers, while maintaining high positioning accuracy in 3D. To achieve this purpose, large inter-element spacing approximately equal to seven wavelengths of sound in air (60 cm) was selected, since the spacing mainly determines the source positioning accuracy for methods based on time difference measurement. Low inter-element distances provide small source to sensor propagation path difference between neighboring system channels, resulting in bigger time delay measurement errors. The initial 60 cm spacing between sensors was selected as a starting point, while later in Section 4.2 it will be optimized to achieve more compact design of the system. As the system is intended for open space measurements, it is expected that the PD source will always be located at least 1 m away from the sensor array. Based on this assumption, a plane wave model has been used in detection of the source position. Three sensors are the minimum amount that can detect objects in a 3D space. Such an approach allows to detect the spatial position of the source, but the observation direction (forward or backward) must be known a priori. In order to obtain the observation direction, an additional sensor must be used. However, in this research three sensors were considered as sufficient.

According to the measured time difference between different ultrasonic channels, the angular direction to the discharge source can be reconstructed in two orthogonal planes from receivers $R_{1}-R_{2}$ and $R_{1}-R_{3}$. As the signals are non-stationary the best way for the time difference determination is the cross-correlation method.

The implementation of the binaural approach for measurements of the position of acoustic noise source in air meets some problems. The ultrasonic waves in air possess quite essential attenuation, while the ultrasonic transducers possess particular frequency bandwidth, sensitivity and directivity. All of these parameters need to be optimized in order to cover required monitoring area. Finally, the general structure of the localization system should meet required accuracy. This includes number of transducers, orientation, position, spacing between sensors etc. The proposed ultrasonic discharge source localization method is based on the time-of-flight difference measured by two pairs of sensors situated on two perpendicular axes ($R_{1}-R_{2}$ and $R_{1}-R_{3}$, see Figure 2). Each pair of the sensors enable to determine a set of directions in 3D where the source generates the delay time measured between either the sensors $R_{1}-R_{2}$ or $R_{1}-R_{3}$. The cross-section of these two sets of 3D directions determines the direction to the true position of the PD source.

**Figure 6:**
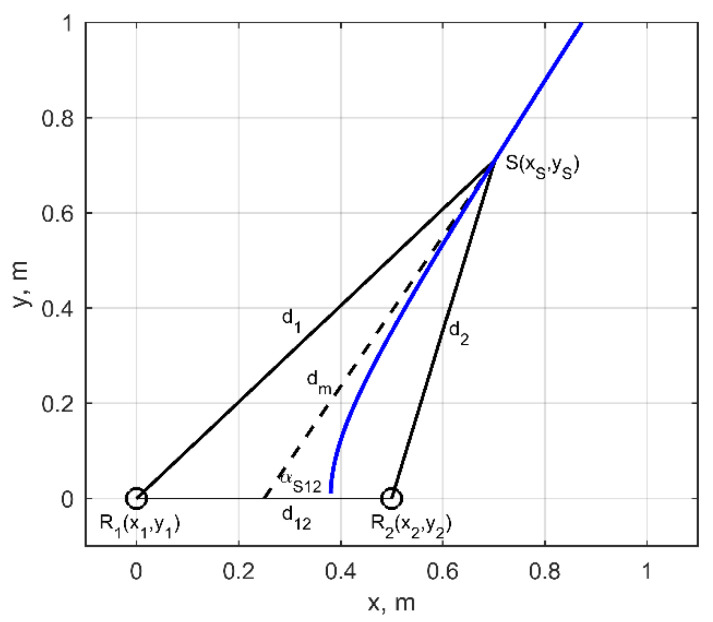
Graphical representation of the 2D approach when the source is located 6 m away in front and 4 m right side.

**Figure 7:**
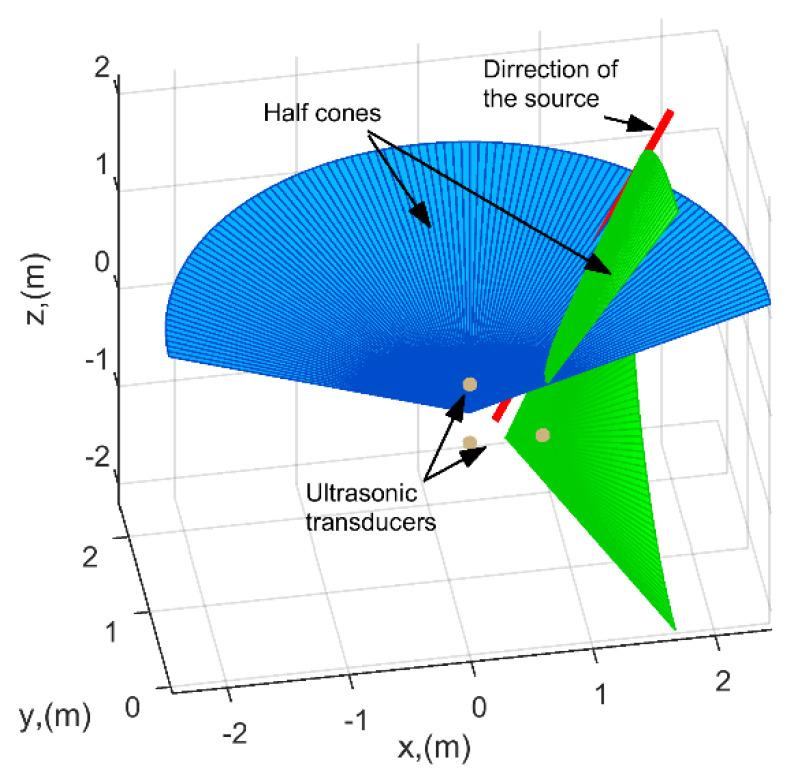
Two cones determining direction to the expected source of acoustic noise.

Let assume that the first pair of the transducers *R*_1_(*x*_1_,*y*_1_,*z*_1_) and *R*_2_(*x*_2_,*y*_2_,*z*_2_) is situated on the *x*-axis with particular distance between them (pitch) *d*_12_ = (*x*_2_ − *x*_1_). If the measured time difference between arrival times of the signal from the sources S to the receivers $R_{1}$ and $R_{2}$ is $\Delta t_{12}$, then the difference of corresponding sound propagation paths can be expressed as:

(13) $$D_{S12}=d_{1}-d_{2}=\Delta t_{12}\cdot c$$

where $d_{1}$, $d_{2}$ are the unknown distances from the source to the receivers $R_{1}$, $R_{2}$; c is the ultrasound velocity in air. The direction to the source from the middle point between transducers is defined by the median of the triangle $R_{1}SR_{2}$ going through vertex S. For better understanding this triangle in Figure 6 is presented for the case when the source is situated in *x*O*y* plane. In 3D space the directions forms a cone (green cone in Figure 7) with vertex angle:

(14) $$\alpha_{S12}=\arccos\left(\frac{d_{m}^{2}-d_{2}^{2}+d_{12}^{2}/4}{d_{m}\cdot d_{12}}\right),$$

where $d_{m}=\sqrt{2\cdot \left(d_{1}^{2}+d_{2}^{2}\right)-d_{12}^{2}}/2$ is the length of the median. It can be shown that at sufficiently large distances the angle $\alpha_{12}$ is dependent only on path difference $D_{S12}$ and distance between transducers $d_{12}$, hence can be calculated according:

(15) $$\alpha_{S12}=\arccos\left(\frac{D_{S12}}{d_{12}}\right).$$

The delay time Δ*t*_12_ can be determined using cross-correlation between signals measured by the receivers $R_{1}$ and $R_{2}$:

(16) $${\Delta t}_{12}=\arg\underset{t}{\max}\left[\mathrm{corr}\left(u_{1}\left(t\right),u_{2}\left(t\right)\right)\right],$$

where $u_{1}\left(t\right)$, $u_{2}\left(t\right)$ are the signals recorded by receivers $R_{1}$ and $R_{2}$. As consequence the beam path difference $D_{S12}=\Delta t_{12}\cdot c$ can be estimated and an angle $\alpha_{S12}$ (Figure 6) can be determined by using Equation (16). In general, such a method gives the direction of plane wave arrival. The blue line and dashed black line in Figure 6 represent the positions of the source according to spherical and plane wave approaches respectively. It can be seen that the essential difference between plane wave and spherical waves can only be seen at distances less than 0.7 m. At larger distances the solution of both methods asymptotically approaches each other.

Another pair of receivers, $R_{1}\left(x_{1},\;y_{1},\;z_{1}\right)$ and $R_{3}\left(x_{3},\;y_{2},\;z_{3}\right)$, situated in vertical direction (along z axis), enable us to determine the angle $\alpha_{S13}$ by creating another cone $C_{13}$ (blue cone in Figure 7) with the vertex in the middle point between the vertical transducers and the axis coinciding with line connecting these receivers. Note that for the better understanding only halves of cones are presented in Figure 7. The cross-section of these two cones give the 3D direction to the source. In general, the cross-section gives two directions: one in the forward direction and one in the backward. However, if the observation direction is known, the correct one can be easily selected. Otherwise, another pair of transducers is required.

In order to determine the direction corresponding to cross-section of these two cones the arbitrary selected distance R essentially longer comparing to the distances between receivers was introduced. In this case R was set to 10 m. Then the spatial points $P_{12}\left(x_{12},y_{12},z_{12}\right)$ determining the directions to noise source according to the cone $C_{12}$ can be defined by:

(17) $$x_{12}=\frac{x_{2}+x_{1}}{2}R\cdot \cos\left(\alpha_{12}\right),\;y_{12}\left(\Theta_{x}\right)=R\cdot \sin\left(\alpha_{12}\right)\cdot cos\left(\Theta_{x}\right),\;z_{12}\left(\Theta_{x}\right)=R\cdot \sin\left(\alpha_{12}\right)\cdot \sin\left(\Theta_{x}\right),$$

where $\Theta_{x}$ is the angle around the *x* axis. In similar way the spatial points $P_{13}\left(x_{13},y_{13},z_{13}\right)$ determining the directions according to the cone $C_{13}$ can be defined by:

(18) $$x_{13}\left(\Theta_{z}\right)=R\cdot \sin\left(\alpha_{13}\right)\cdot \sin\left(\Theta_{z}\right),\;y_{13}\left(\Theta_{z}\right)=R\cdot \sin\left(\alpha_{13}\right)\cdot \cos\left(\Theta_{z}\right),\;z_{13}=\frac{z_{3}+z_{1}}{2}+R\cdot \cos\left(\alpha_{13}\right),$$

where $\Theta_{z}$ is the angle around the *z* axis. Then the direction approximately corresponding to the line of the cross-section of cones $C_{12}$ and $C_{13}$ can be determined by finding the angles $\Theta_{x,min}$ and $\Theta_{z,min}$ at which the distance between pints $P_{12}$ and $P_{13}$ is minimal. The minimum can be determined according:

(19) $$\begin{array}{l} \left[\Theta_{x,min};\Theta_{z,min}\right]\\ =\arg\;\underset{\Theta_{x},\Theta_{z}}{\min}\left\{\left[\sqrt{{\left(x_{12}-x_{13}\left(\Theta_{z}\right)\right)}^{2}+{\left(y_{12}\left(\Theta_{x}\right)-y_{13}\left(\Theta_{z}\right)\right)}^{2}+{\left(z_{12}\left(\Theta_{x}\right)-z_{13}\right)}^{2}}\right]\right\}. \end{array}$$

The spatial angles showing to the direction of the noise are determined according:

(20) $$\Theta_{zOy}=\arctan\left[\frac{y_{12}\left(\Theta_{x,min}\right)-y_{0}}{x_{12}\left(\Theta_{x,min}\right)-x_{0}}\right],\;\Theta_{xOy,z}=\arctan\left[\frac{z_{12}\left(\Theta_{x,min}\right)-z_{0}}{\sqrt{{\left(x_{12}\left(\Theta_{x,min}\right)-x_{0}\right)}^{2}+{\left(y_{12}\left(\Theta_{x,min}\right)-y_{0}\right)}^{2}}}\right],$$

where (*x*_0_,*y*_0_,*z*_0_) are the coordinates of observation point. It was accepted that the distance along *z* axis is *z* = 0. It is more reliable to use *Θ*’*_x_*_O*y*_ and *Θ*’*_x_*_O*y*,*z*_ angles for the further calculations. They show the direction of the source of the acoustic noise in a horizontal and vertical planes and can be determined according:

(21) $$\Theta \prime_{xOy}=90{}^{\circ}-\arctan\left[\frac{y_{12}\left(\Theta_{x,min}\right)-y_{0}}{x_{12}\left(\Theta_{x,min}\right)-x_{0}}\right],\;\Theta \prime_{xOy,z}=90{}^{\circ}-\arctan\left[\frac{z_{12}\left(\Theta_{x,min}\right)-z_{0}}{\sqrt{{\left(x_{12}\left(\Theta_{x,min}\right)-x_{0}\right)}^{2}+{\left(y_{12}\left(\Theta_{x,min}\right)-y_{0}\right)}^{2}}}\right].$$

### 4.2. Experimental Verification of the Proposed Method

In the following chapter, the technique proposed in previous section is verified and optimized with the appropriate experiments. The objectives of the experimental investigation are as follows:

- To determine the correlation strength between signals received by sensors *R*_1_, *R*_2_, *R*_3_ and hereby to investigate the general detectability of the acoustic source.
- To estimate the error and the accuracy of the ultrasonic source localization technique.
- To optimize the pitch between neighboring sensors and to investigate performance of the system in the case of different distance between ultrasonic sensors.

In order to meet these objectives, two measurement set-ups were implemented.

**Figure 8:**
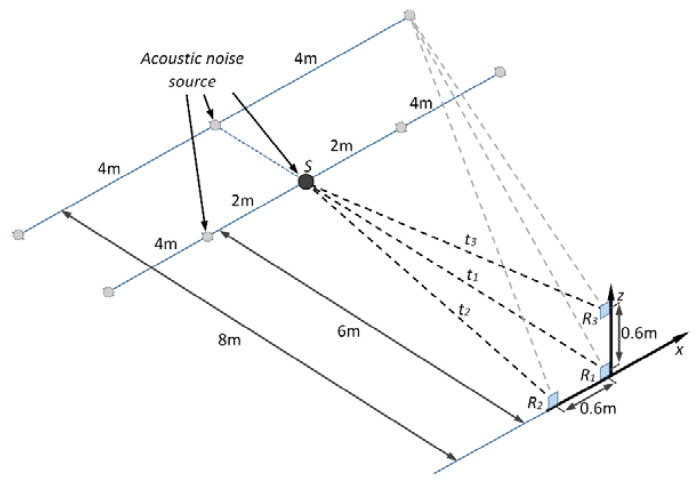
The graphical illustration of the experimental set-up used for detectability.

**(Set-up I).** The aim of this set-up was to perform experiments for discharge signal detectability evaluation and estimation of error and accuracy of discharge source localization technique. The experimental set-up of the measurements with the positioning of the transducers and partial discharge (acoustical noise) source is presented in Figure 8. The experiments were performed with the initial on-axis distance (pitch) between receiving ultrasonic transducers $R_{1}-R_{2}$ and $R_{1}-R_{3}$ equal to 60 cm. The location of the source *S* was changed. The coordinates of the source *S* are summarized in the Table 3.

As it can be seen from the Table 3, at first the needle–needle electrode partial discharge simulator *S* was placed straight in the observation direction at distance of 6 m. Then the direction of the source was changed along the *x* axis to the left (negative *x* direction) and to the right (positive *x* direction) from 2 m to 4 m. At the second stage the experiment was repeated with the initial distance of 8 m. This time, the direction of the source was changed once for each side (left and right) by moving the source by 4 m along *x* axis.

The reception of the emitted acoustic noise signal was performed by using commercial low frequency open-structure ultrasonic transducers MA40S4R produced by Murata Manufacturing Co., Ltd. (Kyoto, Japan). At each of the measurement position, the signals were recorded with three Murata sensors. Then the correlation between the signals was evaluated and the source positioning error was calculated to determine the accuracy of detection.

**Figure 9:**
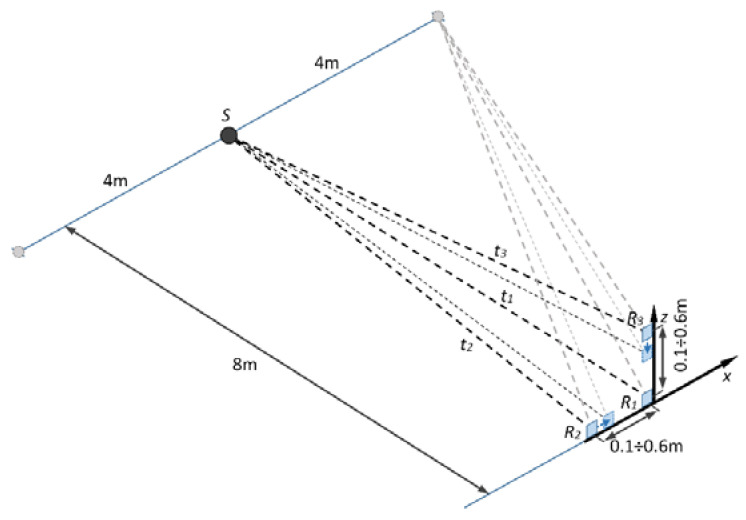
Positioning of the ultrasonic transducers during assessment of least on-axis distance between transducers.

**(Set-up II).** The second experimental set-up was used for inter-element distance (pitch) optimization between sensors in order to optimize the design of the system. At the experiment, the on-axis distance (pitch) between ultrasonic transducers was gradually changed from 50 cm down to 10 cm and with decrement of 10 cm and the determination of partial discharge source position was performed, according to the set-up presented in Figure 9. The measurements were performed in case the partial discharge source is placed within the distance of 8 m away from the receivers straight in the observation direction. After that the partial discharge source was moved away for 4 m to the left and right and the measurements were repeated. In this case, the positions of the source *S* correspond to references 6, 7 and 8 in Table 3. At each measurement position, the signals with all three sensors were collected and source positioning error was estimated in order to determine the minimal on-axis sensor distance at which the PD source can be reliably detected and localized.

### 4.3. Results and Discussion

**Figure 10:**
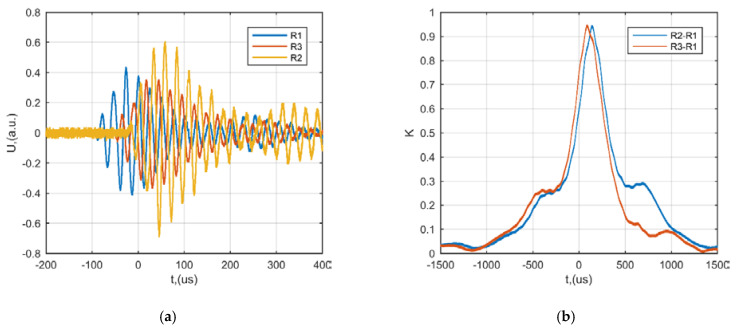
The example of the waveforms and cross-correlation between neighboring channels in case the source S is located straight in the observation point 8 m away along the *y* axis: (**a**) example of waveforms received by $R_{1}$, $R_{2}$, $R_{3}$ sensors, (**b**) correlation between signals in case of receiver pairs $R_{2}-R_{1}$ (blue) and receiver pairs $R_{3}-R_{1}$ (red).

The first measurement set-up (Figure 8) was employed in order to investigate the performance of the proposed measurement system and to identify the possibility to localize the source of acoustic noise by means of cross-correlation between neighboring system channels. The waveforms of the received by $R_{1}$, $R_{2}$ and $R_{3}$ sensors when the source S is located straight in the observation direction at distance 8 m along the *y* axis (position 6 in Table 3) are presented in (Figure 10a).

From the results presented above it can be noticed that time delay between channel $R_{1}$ (blue) and $R_{2}$ (yellow) is ∆*t* = 84.2 µs. Meanwhile the delay between $R_{1}$ (blue) and $R_{3}$ (red) is ∆*t* = 43.4 µs. The correlation strength of the received signals was further investigated. It can be seen that correlation between signals is close to 1 for both receiver pairs $R_{2}-R_{1}$ and $R_{3}-R_{1}$ (Figure 10b), hence the source can be reliably detected.

**Figure 11:**
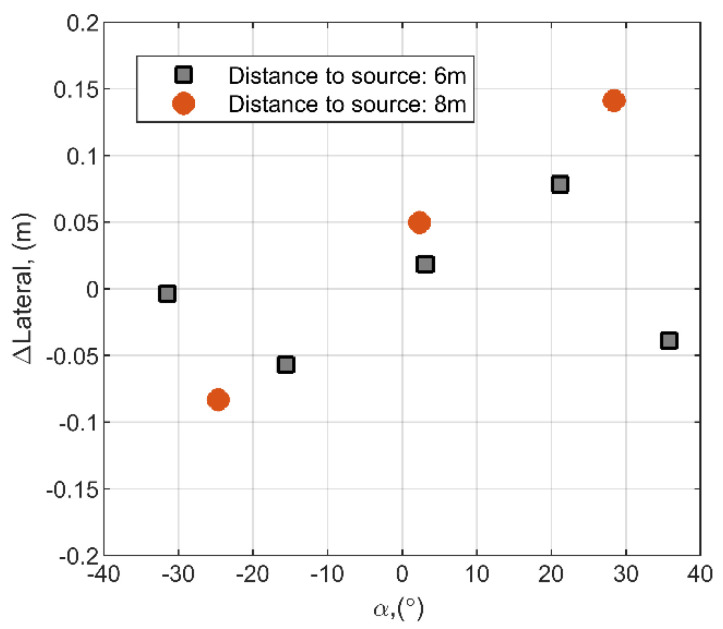
Absolute lateral error versus observation angle *α* at all mentioned positions of the source from Table 3.

Later, the discharge source positioning error was investigated at the certain source positions as it was presented in the first set-up (Figure 8). The discharge source positioning error depends on the observation angle *α* and distance to the sensor and can be expressed as:

(22) $$\Delta Lateral=\left(\alpha_{meas}-\alpha \right)\cdot R_{s},\;R_{s}=\sqrt{x_{s}^{2}+y_{s}^{2}},$$

where *α*_*meas*_—is experimentally measured observation angle, *α*—actual observation angle. The experimentally obtained absolute lateral error dependence on observation angle *α* is summarized in Table 4 and presented in Figure 11.

It can be observed that the distance and the angle dependent lateral error in all cases does not exceed more than 0.15 m. While the works presented by other research groups demonstrate lateral source positioning errors of 5% [32], in this case the estimated lateral error is about 1.3% at 8 m. At such a distance, the experimental time delay between system channels $R_{1}$ and $R_{2}$ is ∆*t* = 84.2 µs at inter-element distance of 60 cm. It can be expected that at 30 m distance, which was used by other authors, the delay would be ∆*t* = 17.5 µs, which is still an easily measurable value.

**Figure 12:**
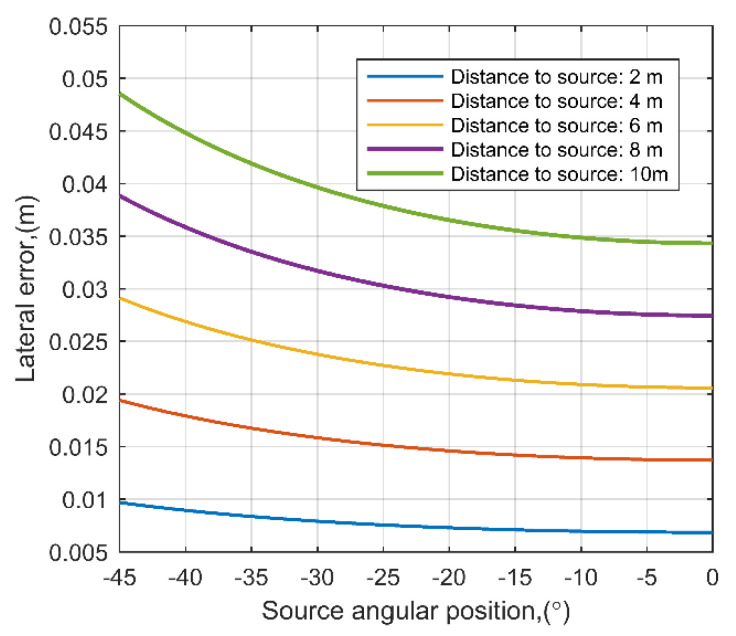
The analytically estimated lateral error dependence on source angular position and distance in case of time measurement error of ±1 µs.

Additional analytical calculations were performed to assess the influence of time difference estimation error to the lateral positioning of the source. The aim of such estimation was to evaluate how the time delay measurement errors influence the positioning accuracy at different angles and source positions. In this calculation it was assumed that the time delay measurement error is fixed to ±1 µs. The equations in Section 4.2 were used to estimate the lateral source position at different source distances and angles. The results of analytical calculations are presented in Figure 12. It can be observed that time measurement error of ±1 µs gives distance and angle dependent lateral error, which is in range of 0.006 m to 0.05 m. This may add significant error at distances larger than 20 m. It was estimated that at 30 m, the lateral error can be up to 0.14 m. On the other hand, time of flight measurement based on cross correlation allow to measure the delay within sub-microseconds in case of sufficient signal sampling and high signal correlation. For example, in the same scenario, the ±0.5 µs time measurement error would provide a lateral source positioning error up 0.07 m at 30 m.

**Figure 13:**
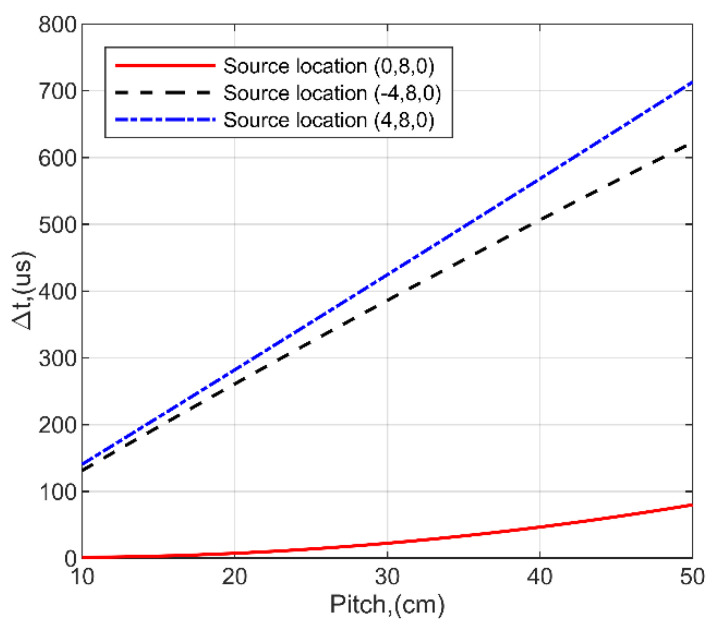
The obtained time differences Δ*t* versus pitch. The source is 8 m away in front (red) and 4 m to the right (blue) or left (black) sides.

The second experimental set-up (Figure 9) was implemented with different inter-element distances between neighboring sensors starting from 50 cm with decrement of 10 cm order to optimize the design of measurement system. The main purpose is to find the least distance of the receivers, at which source of acoustic noise is still detectable. The obtained time differences ∆*t* versus pitch are presented in Figure 13. In which case, when the source is straight in the observation direction 8 m away along *y* axis with a pitch of 20 cm, the time delay is ∆*t* = 2.77 µs. When source is shifted 4 m to the left (Table 3, Pos.7), the time delay increases significantly to ∆*t* = 132 µs. Similar results are obtained after source shifting 4 m to the right (Table 3, Pos. 8), where the time delay is ∆*t* = 139 µs. It means that the side position of the source is more appreciated for detectability. In contrast, the results demonstrate that the source position straight in the observation direction is most challenging to detect, as the signal propagation paths are relatively close to each other and the time difference is mainly determined by the pitch between sensors. This is especially significant when the source in close proximity to the sensors. It was estimated, that the source can be reliably located in case of 20 cm pitch between the sensors, as it provides ∆*t* = 2.7 µs delay between neighboring channels at 8 m distance, what is a measurable value. In the case of a 10 cm pitch, the delay between the sensors at central source position is equal to ∆*t* = 1.34 µs. However, in order to increase the reliability of the measurements in close proximity to the source, the 20 cm pitch was selected for system implementation. It can be calculated that the time delay between sensors $R_{1}$ and $R_{2}$ is ∆*t* = 1.94 µs at 30 m distance and inter-element spacing of 20 cm. This demonstrates that the system can be used for distances up to 30 m. On the other hand, source detection at large distances will require more sensitive reception, higher signal gain and additional noise suppression approaches.

## 5. Recognition of Faulty HV Asset

**Figure 14:**
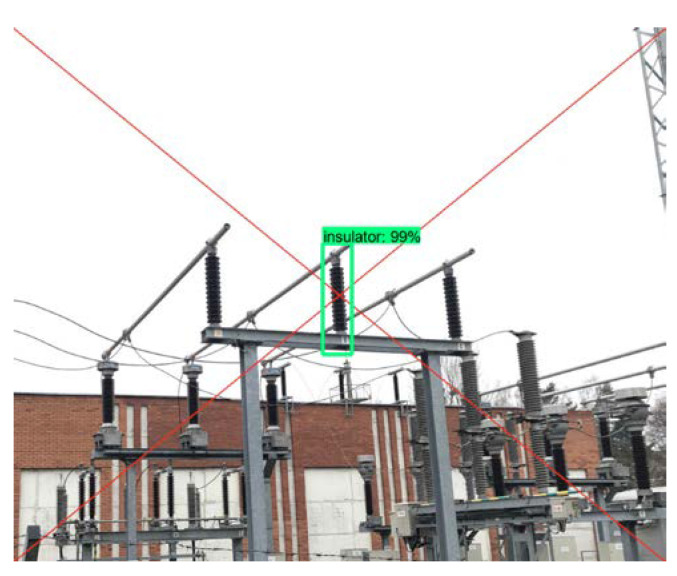
The final output image of the proposed discharge detection system, showing the suspicious element in the center of the image. Deep learning methods are used to determine the origin of the object.

The proposed discharge detection and localization system incorporates an optical camera which is controlled from ultrasonic measurement data by means of pan-tilt servo manipulator (see Figure 1 and Figure 2). The output of ultrasonic signal processing algorithms presented in previous chapter is an angular position of the PD source in horizontal and vertical planes. Such data can be fed to pan-tilt motor, to steer the camera to the desired spatial position. As the camera is positioned in front of the detected discharge source, an optical image is being recorded for further processing. Typically, in such image, many bushing insulators are present, depending on the mounting position of the device in the transformer substation. So it’s important to distinguish a single bushing insulator that possess a risk of discharge and mark it to the end user. In order to detect and emphasize single suspicious discharging insulator, deep learning convolutional neural networks (CNN) are applied and embedded to the system. Usually, if the spatial location of acoustic source is correctly determined by ultrasonic methods described in previous chapter, the discharging source should be located somewhere at the center of an image. So, it is assumed here that the central area of the image most likely will contain the view of discharging asset. Hence, the convolutional neural networks are used to process the area around center point of the image and detect the assets of bushing insulators. To achieve this purpose, as a first step. a small area around the center of an image is cropped and provided to a CNN object detector. If none of the objects are detected, then the size of an area is increased until the object detector finds at least one insulator. The final output is the original image captured by optical camera with boundary of single insulator. The CNN object detector is pre-trained to detect high voltage transformer bushings. The detector is implemented by using Faster R-CNN convolutional neural network, which uses a region proposal network (RPN) for feature map estimation and Fast R-CNN as a detector [35]. Faster R-CNN is an evolution of its predecessor, Fast R-CNN, and proposes anchors that most likely contains objects to generate region proposals instead of selective object search. The CNN model was trained using TensorFlow GPU v1.9 object detection API on NVIDIA Geforce GTX 1060 with CUDA v9.0.176 and cuDNN v7.0. In total, 430 images of transformer bushing insulators were labelled and used in training dataset, while the testing dataset was compiled from images that were acquired from different transformer stations across Lithuania. The model was trained in 150k epochs (batch size 1) achieving average classification loss of 0.04 and mean average precision (mAP) of 91.31%. The example of single discharging bushing insulator which was detected using Faster R-CNN is presented in Figure 14.

Object detection with CNN is used here for two purposes: first, the single discharging asset is recognized in the image, which serves as an output of the detection device; second, if none of the assets are recognized in the image, the measurement is repeated as this is treated as the measurement error. Hence CNN acts as an additional evaluation loop to avoid false alarms.

## 6. Conclusions

The ultrasonic non-invasive approach for detection, localization of the partial discharge (PD) that is designed to assess dielectric conditions of connectors of bushing insulators was proposed. The proposed solution uses machine learning, ultrasonic signal processing and deep learning methods to detect and localize the source of PD. At the first stage ML methods act as a firewall to filter acoustic noise signals that are captured by the system and differentiate between actual PD signals and surrounding noise. Then the binaural methods are used for PD source localization which exploits the time difference between arrival time of the same discharge signal received by different ultrasonic sensors. Based on these methods, the angular direction of PD source is reconstructed in 2D and 3D space with an average lateral positioning error of 0.1 m. Finally, ultrasonically estimated positions of PD source are fed to pan-tilt servo manipulators to steer the optical camera to the detected PD source position. Then the recognition of the discharging asset is performed by using deep learning convolutional neural networks which identifies single discharging HV assets.

The proposed ultrasonic PD localization and detection technique was verified and optimized with the appropriate experiments. At first, ML methods were evaluated for separation between discharge signals and surrounding noise. It was estimated, that two signal features (kurtosis and difference absolute standard deviation) holds the most variance and are most appropriate for determination of the noise origin. Among the different ML classifiers, it was found that RBF-SVM is the one that shows the best sensitivity and specificity as it provides a non-linear classification boundary. A separate set of the experiments were performed with the different positions of the discharge source and inter-element distances between sensors *R*_1_, *R*_2_, *R*_3_ with the aim to investigate the detectability, lateral error and optimal spacing between sensors. The investigations demonstrated a strong correlation between PD signals and the lateral error of source positioning was estimated to be up to 0.1 m. The inter-element spacing optimization experiment demonstrated that 20 cm pitch between the sensors is optimal in order to reliably detect PD source at 8 m distance as it provides 2.7 µs delay between neighboring channels. It was shown that the inter-element spacing plays a significant role for sources that are positioned in front of the detector, while the sources at some angles are easier to detect due to longer signal propagation paths. As a result, a smaller pitch such as 10 cm may be insufficient to detect the PD at certain positions. Finally, the deep learning neural networks was implemented which are capable to detect single suspicious discharging assets in optical images based on the spatial PD positions determined by ultrasonic methods. As a result, the output of the proposed system is an intuitive discharge visualization in a real-scene environment.

As the results presented in this article showed quite good performance both in classification of discharge source and localization of it in 3D space, a logical next step would be to apply these methods in an actual substation with pre-identified faulty insulators. This would include additional challenges as more significant signal reverberations, changing environmental conditions, increased noise levels and reduced PD signal energies. As a result, one might expect to obtain larger positioning errors and a lower classification accuracy. On the other hand, the ML techniques used in this approach can improve on each measurement, while receiving more and more data describing the PD signals. With the advent of ML methods, better understanding of the PD signals may subsequently lead to an increased overall detection and localization accuracy on the long-term perspective.

## Figures and Tables

**Table 1:** *T*-test and Pearson correlation values between discharge and noise datasets.

	Mean Absolute Value, *M*_AV_	Variance, *v*	Simple Squared Integral, *S*	Kurtosis, *K*	Root Mean Square, *R*	Average Amplitude Change, *A*	Difference Absolute Standard Deviation, *D*	Modified Mean Absolute Value, *w_i_*	Maximum Fractal Length, *M*_F_
*h_0_*	0	1	1	1	1	0	1	0	1
*p*-value	0.069	0.017	0.017	4.5 × 10^−22^	0.016	0.052	0.03	0.267	0.038
*corr*	−0.149	−0.19	−0.19	0.08	−0.14	−0.14	−0.13	−0.02	−0.04

**Table 2:** Comparison of sensitivity and specificity of three classifiers in the case of six and two feature vectors used for classification.

	Six Feature Vectors		Two Feature Vectors	
	Sensitivity:	Specificity:	Sensitivity:	Specificity:
*RBF SVM*	100%	95%	95.6%	92.5%
*Naïve Bayes*	93.3%	95%	93.3%	92.5%
*LDA*	100%	95%	93.3%	90%

**Table 3:** The coordinates of the source *S* used in the experiments with set-up I and set-up II.

Position no.:	*x*, (m):	*y*, (m):	*z,* (m):
1	0	6	0
2	−2	6	0
3	−4	6	0
4	2	6	0
5	4	6	0
6	0	8	0
7	−4	8	0
8	4	8	0

**Table 4:** Absolute lateral error values dependent on special source position.

No.:	Source Position (*x*,*y*,*z*), (m):	Mean Angle (*Θ*’*_x_*_O*y*_), (°):	Mean Angle (*Θ*’*_x_*_O*y*,*z*_)*,* (°):	Lateral Error, (m):
1	(0,6,0)	3,3	–1,8	0.018
2	(–2,6,0)	–16,1	–2,2	0.056
3	(–4,6,0)	–31,5	–2,3	0.0035
4	(2,6,0)	21,9	–1,4	0.078
5	(4,6,0)	35,5	–0,63	0.038
6	(0,8,0)	2,7	–1,43	0.05
7	(–4,8,0)	–25,2	–1	0.083
8	(4,8,0)	29,3	–0,53	0.14

## Data Availability

Data sharing not applicable.

## Abbreviations

AUC	area under curve
CNN	convolutional neural network
DBSCAN	density based spatial clustering of applications with noise
DL	deep learning
HV	high voltage
LDA	linear discriminant analysis
ML	machine learning
NB	Naïve Bayes
PCA	principal component analysis
PD	partial discharge
RBF	radial basis function
ROC	receiver operating curve
RPN	region proposal network
SVM	support vector machines
t-SNE	t-distributed stochastic neighbor embedding
